# Conservative management of early-stage endometrial cancer for fertility preservation: a survey study among Swedish gynecologists and gynecological oncologists

**DOI:** 10.1038/s41598-023-32911-y

**Published:** 2023-04-11

**Authors:** Stavros I. Iliadis, Pietro Gambadauro

**Affiliations:** 1 Department of Women’s and Children’s Health, Uppsala University, 751 85 Uppsala, Sweden; 2 Department of Learning, Informatics, Management and Ethics (LIME), Karolinska Institutet, 171 77 Stockholm, Sweden; 3 Res Medica Sweden, 753 15 Uppsala, Sweden

**Keywords:** Infertility, Endometrial cancer

## Abstract

Conservative management of endometrial cancer (CMEC) is viable for women with early-stage disease wishing to preserve fertility, but there is poor knowledge regarding clinicians’ attitudes towards treatment or guidelines adherence. This 55-item survey study investigated CMEC-related experience, practice and attitudes among clinically active Swedish gynecologists and gynecological oncologists, focusing on reproductive eligibility criteria. The survey consisted of a general and two specific subsets, selectively delivered to clinicians active in infertility (subset A) and endometrial cancer (subset B) care. Answers from 218 clinicians were included. More than half agreed on CMEC whereas only 5% explicitly disagreed. The majority supported a fertility work-up to substantiate reasonable chances to pregnancy and live birth. Most disagreed about CMEC in case of previous unsuccessful fertility treatments, while more than 1/3 disagreed about CMEC in known fertility problems, recurrent miscarriages or previous children. Over 50% of respondents in subset A (n = 107) found it applicable with fertility investigations such as ovarian reserve testing or, in case of male partner, semen analysis. Respondents in subset B (n = 165) agreed on items based on existing recommendations regarding the oncological management of CMEC, including the use of continuous progestins, hysteroscopic resection of macroscopic lesions, control biopsy with curettage or hysteroscopy after 6 months of treatment, pursuing pregnancy as soon as possible after complete response, and performing a hysterectomy once live birth is achieved. While many clinicians were familiar with CMEC, the overall experience is limited. Fertility specialists seem less involved than oncologists in patient care but there is broad support for fertility-related eligibility criteria.

## Introduction

Endometrial cancer (EC) is the most common gynecologic cancer in Sweden as well as worldwide and the sixth most commonly occurring malignancy among women^1,2^.

Hysterectomy, with bilateral oophorectomy, is usually effective for EC, with limited drawbacks for most affected women, who typically are postmenopausal^1,2^. A minority of cases, however, occur during reproductive age and 5% of women are younger than 40 years old at the time of diagnosis^1^. In such cases, the standard surgical treatment means absolute uterine factor infertility. To preserve the reproductive potential of these women, conservative management of endometrial cancer (CMEC) is offered internationally^3–5^. The treatment usually requires the administration of oral or intrauterine progestins, sometimes after hysteroscopic resection of the malignancy, to achieve a complete response and offer the chance for pregnancy and live birth before eventually completing the treatment with standard surgery^3^.

Literature supports CMEC for women with early-stage (clinical stage I, grade I) endometrioid malignancy who wish fertility preservation^3^. In fact, progestins induce a complete response in most of such cancers^3,6^. Besides, promising chances of live birth have been estimated in a recent meta-analysis, such as 20.5% in the overall group of women undergoing CMEC, 30.7% among women younger than 36, and 42.4% when patients are followed-up for at least 36 months^3^.

Nevertheless, the choice between the standard oncological treatment and CMEC is challenging. The reproductive results of individual studies are heterogeneous and long-term oncological outcome data are lacking^3^. Clinicians may therefore need to individualize their approach but at the same time, most of them only have limited clinical caseloads^4^.

Although recommendations and consensus statements have been published during the last decade^6–8^, there is poor knowledge regarding clinicians’ attitudes towards CMEC or their adherence to recommended practice. A study among young gynecological oncologists in Europe reported that, despite similar diagnostic approaches regarding CMEC, uncertainties and disagreements on several management-related matters are common, and the authors called for guidelines to achieve consistent practice^4^. Interestingly, even non-clinical factors such as caseload and setting may influence attitudes and practice regarding fertility preservation for gynecological malignancy, according to a survey among American gynecological oncologists^9^. More recently, French gynecological surgeons and fertility specialists reported lack of confidence in their knowledge on CMEC. However, most surgeons informed potential candidates about fertility preservation^10^.

Despite consensus regarding the oncological criteria for CMEC, existing recommendations are affected by specific knowledge gaps concerning which reproductive prognostic criteria should define treatment eligibility^7,8^. In the most recent Swedish care program for endometrial cancer, for instance, the topic of fertility sparing treatment is only briefly addressed with a focus on oncological eligibility and management criteria while in-depth guidance for reproductive professionals is lacking^2^. This is problematic because increasing trends in the clinical use of CMEC notwithstanding, the outcomes in real-life settings may be worse than those reported in literature^3,5^. Consequently, calls have been made for improved interdisciplinary efforts regarding fertility preservation for EC^3,5,11^.

This study aimed to investigate experience, practice and attitudes regarding CMEC for fertility preservation purposes in a national survey among gynecologists and gynecological oncologists in Sweden. A specific objective was to elucidate eligibility criteria for CMEC in relation to the reproductive prognosis. A secondary objective was to evaluate the agreement between local practice and existing recommendations on the oncological management of CMEC.

## Results

Of 242 survey records, 23 were removed because they were empty (n = 9), partial (n = 7) or duplicated (n = 7). After exclusion of one non-clinical respondent, the final sample included 218 participants (median age 45) whose characteristics are summarized in Table 1.

**Table 1:** Characteristics of the study population (N = 218).

Variable	N	%
Gender (N = 216)		
Female	167	77.3
Male	46	21.3
Other	1	0.5
Do not wish to answer	2	0.9
(missing)	(2)	
Professional role		
Specialist	181	83
Resident	37	17
Clinical specialty		
Obstetrics/gynecology	206	94.5
Oncology	10	4.59
Obstetrics/gynecology and other	2	0.91
Basic/specialist training in Sweden		
Only in Sweden	155	71.1
Partially/completely abroad	63	28.9
Main workplace		
County hospital	94	43.2
University hospital	91	41.7
Outpatient care	33	15.1
Engaged in research		
Yes	104	47.9
No	113	52.1
(missing)	(1)	
Region		
Stockholm	40	19.3
Västra Götaland^a^	36	17.4
Skåne^b^	28	13.5
Uppsala	23	11.1
Other^c^	80	38.7
(missing)	(11)	
Active in infertility care (subset A)	107	38.1
Active in endometrial cancer care (subset B)	165	58.7

^a^Gothenburg’s region.

^b^Malmö’s region.

^c^Blekinge (n = 3), Dalarna (n = 6), Gotland (n = 2), Gävleborg (n = 2), Halland (n = 11), Jämtland (n = 3), Jönköping (n = 9), Kalmar (n = 8), Kronoberg (n = 2), Sörmland (n = 4), Värmland (n = 5), Västerbotten (n = 6), Västernorrland (n = 2), Västmanland (n = 2), Örebro (n = 4), Östergötland (n = 11).

Most participants were certified specialists (83%), reported Obstetrics & Gynecology as specialty (94.5%) and worked in one of Sweden’s four major conurbations (61.3%). According to the predefined criteria mentioned in the Material and methods section, 107 participants qualified for subset A (fertility-specific), 165 for subset B (oncology-specific), and 83 for both subsets.

### General question subset

**Figure 1:**
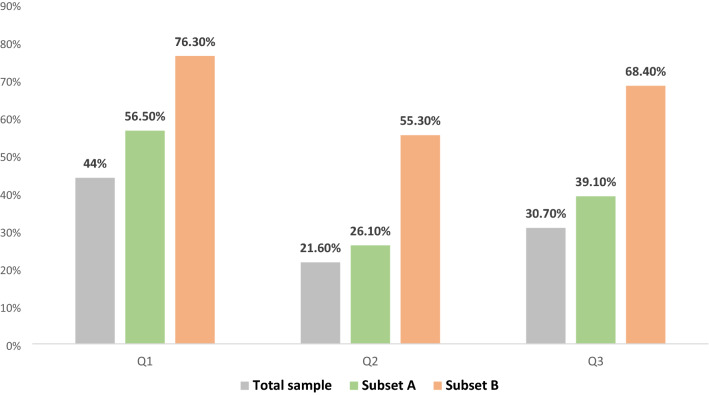
Clinical experience on conservative treatment of endometrial cancer for fertility preservation purposes. Question 1 (Q1): In your clinical work, have you ever been involved in the care of women with endometrial cancer who wished fertility preservation? Question 2 (Q2): In your clinical work, have you ever suggested or offered conservative treatment of endometrial cancer for fertility preservation? Question 3 (Q3): In your clinical work, have you ever been involved in the care of women who underwent conservative treatment for fertility preservation? Subset A: clinicians active in infertility care (N = 107). Subset B: clinicians active in endometrial cancer care (N = 165). The chart illustrates positive answers.

Figure 1 illustrates the clinical exposure to CMEC, overall and among clinicians qualifying for any of the question subsets. The proportion of clinicians reporting experience with CMEC was larger among clinicians working with infertility or oncology compared to the overall sample. This was particularly evident among clinicians who managed endometrial cancer patients. Previous involvement in CMEC was also more frequent among research-active respondents, compared to non-research active peers (χ^2^ test; *P* < 0.05).

Most participants (115/218, 52.8%) agreed on CMEC for motivated women with early-stage disease, 41.7% had no opinion and the remaining 5.5% (n = 12) disagreed and hence were excluded from further questions. Similar proportions of participants agreed with CMEC among those qualifying for question subset A (59/107, 55.1%), subset B (90/165, 54.5%) or both subsets (45/83, 54.2%). Experience of medical education/training outside Sweden and research activity were positively associated with agreement on offering CMEC (χ^2^ test; *P* < 0.05).

Most respondents agreed on performing a fertility workup before CMEC to confirm reasonable chance for spontaneous (n = 129, 69%) or medically assisted (n = 133, 71.1%) pregnancy and childbirth. Most respondents (87.6%) also agreed about requiring a minimum likelihood of pregnancy and childbirth, but there was poor consensus regarding such threshold and 36.8% could not define one. The oldest acceptable age for CMEC was most frequently set at 40 years (48.9%), while 6.5% found age-limits irrelevant. The most frequently chosen upper Body Mass Index (BMI, kg/m^2^)-limit was 29/30 (45.7%), and 15% found BMI-limits irrelevant.

**Figure 2:**
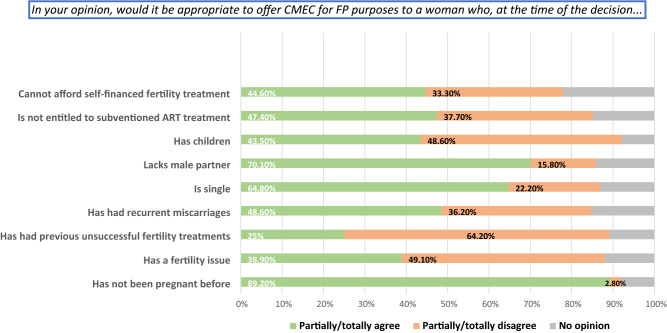
Opinions on fertility-related eligibility criteria for conservative treatment of endometrial cancer. *CMEC* Conservative management of endometrial cancer, *FP* fertility preservation, *ART* assisted reproductive technology.

Figure 2 presents opinions on further demographic and anamnestic eligibility criteria for CMEC. Most respondents disagreed with offering CMEC in case of previous unsuccessful fertility treatments (64.2%), while almost half of them disagreed for someone with children or known fertility problems. More than one third of respondents disagreed on CMEC for persons with recurrent miscarriages or when private or public funding is unavailable, while few saw the lack of partner or being nulligravida as exclusion criteria.

### Fertility-specific question subset

**Figure 3:**
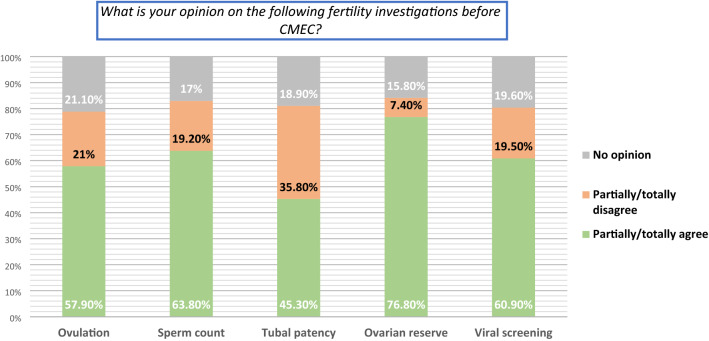
Opinions on fertility-specific investigations upon conservative management of endometrial cancer. *CMEC* Conservative management of endometrial cancer. Question subset A was delivered to clinicians active in infertility care (N = 103). Response rate > 89% for all items. Ovarian reserve assessed with AMH (Anti-Mullerian Hormone) or AFC (Antral Follicle Count).

Additional questions regarding the components of an infertility work-up before CMEC were answered in this subset (Fig. 3). Eligible for these questions were 103 of the 107 clinicians active in infertility care, after exclusion of those expressing disagreement with CMEC in the general questionnaire (n = 4). The response rate exceeded 89% for all items. Most standard investigations were considered applicable to CMEC candidates, with the greatest support for ovarian reserve testing (76.8% agreement) and the lowest for tubal patency tests (36% disagreement). Approximately 15–20% had no opinion on the subset items.

### Oncology-specific question subset

**Figure 4:**
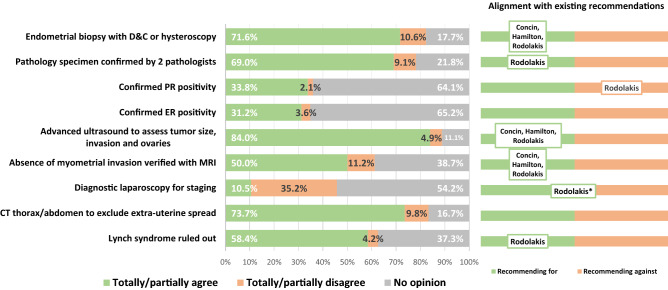
Oncology-specific diagnostic criteria for conservative management of endometrial cancer in the study population as well as in relation to international recommendations and guidelines. *D*&*C* Dilatation and curettage, *PR* progesterone-receptor, *ER* estrogen-receptor, *MRI* magnetic resonance imaging, *CT* computerized tomography. Question subset B was delivered to clinicians active in endometrial cancer care (N = 156). Response rate > 88% for all items. Citations with author names presented within the chart bars to the right refer to published guidelines recommending for/against individual diagnostic criteria. *Recommendation to discuss laparoscopy with the patient^6–8^.

**Figure 5:**
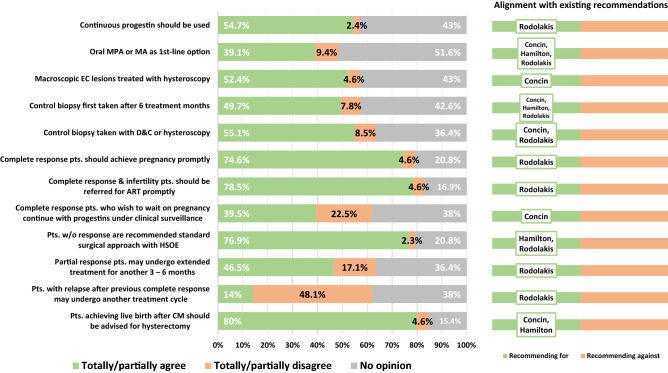
Oncology-specific treatment and follow-up criteria for conservative management of endometrial cancer in the study population as well as in relation to international recommendations and guidelines. *MPA* medroxyprogesterone acetate, *MA* megestrol acetate, *EC* endometrial cancer, *D*&*C* dilatation and curettage, *ART* assisted reproductive technology, *Pts* patients, *w/o* without, *HSOE* hysterectomy and salpingo-oophorectomy, *CM* conservative management. Question subset B was delivered to clinicians active in endometrial cancer care (N = 156). Response rate > 82% for all items. Citations with author names presented within the chart bars to the right refer to published guidelines recommending for/against individual treatment/follow-up criteria^6–8^.

These questions addressed the oncological management of CMEC patients (Figs. 4 and 5) and were delivered to 156 of the 165 clinicians active in EC care, after exclusion of those expressing disagreement with CMEC in the general questionnaire (n = 9). The response rate exceeded 88% for diagnostic items and 82% for treatment/follow-up items. Overall, responses on diagnostic, treatment and follow-up criteria showed consistent agreement with existing recommendations. The lowest support was received by laparoscopic staging (35.2% disagreement) and repeated CMEC in patients with a relapse after complete response (48.1% disagreement). A substantial proportion, however, expressed uncertainty on several items, with more than 50% having no opinion on the need of confirming estrogen or progesterone receptors positivity, laparoscopic staging, and oral progestins as first line treatment.

## Discussion

This study elucidates experience, practice and attitudes regarding CMEC among gynecologists and gynecological oncologists in Sweden. Many clinicians have been involved in the care of women undergoing CMEC and more than half of them agree with the treatment; however, extensive experience is lacking. Differences between medical specialties were observed, with oncologists being more often engaged in fertility preservation questions, as expected since these clinicians primarily counsel patients and decide on cancer treatment. Fertility preservation is often under-prioritized upon cancer treatment and fertility specialists are not always involved in care of this patient group. Indeed, in the present study, many fertility specialists lacked experience and one-third of them had no opinion whether CMEC should be offered to women where indicated. Overall, however, only 5% explicitly disagreed with CMEC, suggesting that support for this novel treatment strategy might increase as local experience, scientific evidence and guidelines become more robust. In a French survey among gynecological surgeons and fertility specialists, approximately half of respondents found it difficult to manage patients regarding CMEC, probably due to lack of confidence in their knowledge^10^. In fact, knowledge scores towards fertility preservation were low in the same study. However, most surgeons still advised patients on CMEC before offering cancer treatment^10^.

This study focused on reproductive prognostic factors and eligibility criteria which, according to the respondents, ought to be considered before CMEC. It has been recommended that CMEC candidates should be referred to fertility specialists because the individual reproductive prognosis is important in decision-making^2,5,7,12^. However, clinical implementation is challenging, and clinicians lack clear guidelines regarding fertility-related eligibility for CMEC^11^. The Ethics Committee of the American Society for Reproductive Medicine states that fertility treatment should be patient-centered and decision-making should be based on the assessment of treatment-related risks and benefits^13^; very low success chances can result in declining medical treatment^14^ and provision of futile therapies may be ethically unjustifiable^13^. Conservative management seems viable for selected women with EC, considering that about 75–80% exhibit a complete response; however, 10–35% of those have disease recurrence, and one-fifth of women achieve live birth. Therefore, guidance on the contribution of fertility specialists in decision-making and counseling seems crucial^3,15,16^.

Many clinicians in our study found that an upper age limit of 40 years would be appropriate, which is in line with findings from the study among French clinicians, where a threshold of 38–40 was suggested^10^. Only 6.5% in the present study would consult for CMEC regardless of age. It should be mentioned that the upper female age limit for publicly funded oocyte and embryo freezing for fertility preservation in Sweden is 40 years^17,18^. In a recent meta-analysis, the highest chance of live birth was observed in studies recruiting women of age 35 or younger^3^, which should be considered upon counseling and selection.

Only 15% of participants found it unnecessary with BMI limits for CMEC while almost half of them indicated an upper limit of 29–30 kg/m^2^ as most appropriate. This could be related to current national practice since public-funded fertility clinics in Sweden apply BMI limits of 30–35 kg/m^2^ for fertility treatments. BMI lower than 35 has been associated with higher remission and pregnancy rates in CMEC in some studies^19,20^, but other results are contradictory^15,16^. Further studies need to investigate BMI limitations in the context of CMEC as well as the possible impact of obesity on disease recurrence and live birth outcomes.

Half of participants in our study would advise against CMEC in case of known subfertility and almost two-thirds would act likewise in case of previous unsuccessful fertility treatments. Moreover, more than two-thirds of respondents would perform a preliminary fertility work-up to confirm reasonable chance for spontaneous or medically-assisted pregnancy. Most clinicians also found a lowest probability threshold for pregnancy and live birth to be a requirement for CMEC. Interestingly, previous meta-analyses indicate that infertility history is associated with higher remission rates^15,16^ and similar pregnancy rates after CMEC^16^. Improved pregnancy rates are usually related to higher chance of remission and the frequent use of assisted reproductive technology (ART)^3,16^. Nevertheless, a history of unsuccessful treatments may be considered as a negative prognostic factor^21^. Counterintuitively, almost half of respondents would advise against CMEC in women with children. This finding should be interpreted considering that only childless single women and couples without common children are entitled to publicly funded treatment in Sweden.

Most respondents would use standard fertility investigations to facilitate patient selection. Many participants found ovarian reserve as a useful criterion upon CMEC. However, a diminished ovarian reserve cannot safely distinguish between a pathologic or expected decline in fertility and does not necessarily equate with conception inability^22^. On the other hand, ovarian reserve testing predicts gonadotropin stimulation response and can be useful upon ART. On a related note, one-third would not assess tubal patency; a finding attributed to the broad use of ART after CMEC. However, information on tubal patency may allow for counseling of women seeking spontaneous pregnancies after CMEC. Interestingly, 15–20% of study participants expressed an inability to decide on the criteria’s usefulness upon CMEC, probably elucidating a lack of fertility-oriented guidelines regarding EC treatment for fertility preservation purposes.

The study shows agreement between the opinions of most Swedish clinicians on the oncological management of CMEC cases and existing guidelines^6–8^. Regarding cancer-related work-up criteria, most respondents agreed on the operative and imaging evaluation strategies of CMEC patients. Nevertheless, there was uncertainty on items having sparse support in guidelines, pointing to evidence gaps that warrant further studies. For example, only one-third would require estrogen- or progesterone-receptor positivity. Data on hormone receptor status within the context of CMEC are sparse and related recommendations are inconclusive^8^.

Similarly, opinions on cancer treatment and follow-up were generally in accordance with existing recommendations. For example, most agreed on continuous progestins, hysteroscopic resection of macroscopic lesions, and control biopsy after six months. However, significant proportions of respondents were unable to express an opinion (15–52%) and specific items appeared controversial. For example, 22.5% disagreed with continuing progestin treatment under surveillance for women who delay their attempts to conceive after complete response, although such strategy is indeed recommended internationally and in Sweden^2,23^.

Most clinicians disagreed on repeating CMEC after disease recurrence, in women with previous complete response. The related evidence is indeed sparse. Disease recurrence is common^3,24^ and long-term effects of treatment postponement are insufficiently studied^3^; on the other hand, available data on hormonal treatment in young women with early-stage EC has shown encouraging results regarding long-term cancer survival^25^. Decision-making should probably weigh in individual benefits and risks and consider the patient’s perspective.

This is the first study to investigate experience and attitudes towards CMEC among clinicians in Sweden, while only few similar studies exist globally. By reaching out to national professional societies, most clinicians in the relevant target group were approached, including fertility specialists. Only one previous study investigated the latter group but did not include gynecological oncologists^10^. Our study used a structured questionnaire, addressed oncological and gynecological aspects of CMEC and was based on current international guidelines and practice, thus allowing for comparisons with existing literature.

The inability to report on response rate and potential non-response bias is a limitation. Despite SFOG and SSGO being the best available platforms for reaching out to potential study participants, the proportion of clinically active members in their mailing lists is unknown. However, it can be hypothesized that the specific topic of this study attracted clinicians managing women with infertility and/or EC and thus being relevant to the objectives. Besides, a previous Swedish study achieved a sample size similar to ours through the same platforms, despite a broader research topic (i.e., attitudes towards hormone replacement therapy)^26^. Similarly, although we lack information regarding the individual experience of the respondents regarding EC or fertility preservation, clinicians working in specialized centers and with specific experience could be over-represented in our study. Almost half of respondents worked at a university hospital or were active in research, and the latter group was more positive towards CMEC, compared to rest of the study population. Nevertheless, that was to some extent in line with the study aims, because groups of highly specialized clinicians are expected to be responsible for CMEC patients in most clinical settings. Regarding the contents of the questionnaire, finally, emerging evidence suggests that novel molecular classifications might aid patient selection for CMEC, particularly in complex cases^27^. This was not addressed in the current study, which largely draws from previous surveys and published guidelines. However, as new promising data are being added to the knowledge base^28^, it would be interesting to further investigate the opinion and attitude of professionals towards these novel molecular criteria.

## Conclusion

This is the first investigation of experience, practice and attitudes regarding the conservative management of early-stage endometrial cancer among Swedish gynecologists and gynecological oncologists. While many clinicians are familiar with the treatment, the overall experience is limited and a significant proportion could not express an opinion on offering conservative management where indicated. Fertility specialists are less involved than oncologists in patient counseling and care but there is support for fertility-related eligibility criteria. Interdisciplinary guidelines and collaboration are essential to provide individualized counseling and optimize care.

## Material and methods

### Design and study population

A national survey study targeting clinically active gynecologists and gynecological oncologists in Sweden was undertaken between May and November 2021. A web-based questionnaire was developed on the REDCap (Research Electronic Data Capture) tool hosted at Uppsala University^29,30^. Invitations with study information and links to the questionnaire were emailed through the mailing lists of the Swedish Society of Obstetrics & Gynecology (SFOG) and the Swedish Society of Gynecological Oncology (SSGO). Most specialists and residents in Obstetrics & Gynecology and Gynecological Oncology in the country are members of these societies. Since no previous surveys have been conducted on the same topic in Sweden, all registered members were invited without a priori sample size calculation. Two reminders were sent out after the initial invitation. The survey was eventually closed once no new responses were incoming and the final sample was benchmarked against a previous online survey on a broader topic (i.e., hormonal replacement treatment after gynecologic cancer) among members of the same two societies^26^.

### Survey structure and questions

The questionnaire comprised 55 questions/statements designed for the purpose of the study. Three question subsets (general, fertility-specific and oncology-specific) were presented to relevant groups of participants according to a predefined branching logic.

One screening item identified clinically active respondents, who were thereafter delivered the general question subset starting with items about socio-demographics (age, gender, region) and professional background (e.g., role, specialty, education, setting, research experience). Experience within the field of CMEC was evaluated with three items investigating previous or current involvement in CMEC for fertility preservation (never/ ≤ 5 times/6–10 times/ > 10 times). The attitude towards CMEC was evaluated by one question asking whether, in the respondent’s opinion, CMEC could be offered for fertility preservation to women with early-stage EC (yes/no/cannot decide). Respondents who disagreed with CMEC could not progress further in the survey. These explored opinions regarding eligibility criteria for CMEC, such as requiring a fertility workup, minimum likelihood of pregnancy and live birth, age and BMI limits, and other factors linked to the reproductive potential of candidates (14 items in total).

Questions in the fertility- and oncology-specific subsets investigated agreement on selected diagnostic or treatment criteria (possible answers; totally/partially agree/disagree, cannot decide). The fertility-specific subset (A) addressed fertility investigations to be performed before CMEC, based on current standards (i.e., assessment of ovulation, ovarian reserve, tubal patency, semen analysis, and viral screening). This subset was delivered to clinicians active in infertility care (i.e., specializing in reproductive medicine or otherwise managing subfertile patients). The oncology-specific subset (B) addressed oncological aspects of CMEC (diagnostics, 9 items; treatment/follow-up, 12 items), with items based on existing recommendations^7,8^ and a previously published survey on the topic^4^. These questions were delivered to clinicians active in endometrial cancer care (i.e., specializing in gynecological oncology/oncological surgery or otherwise managing women with EC).

### Statistical analyses

To describe background characteristics of the study population, descriptive statistics were used. The main outcomes of the study were summarized and presented as valid percentages (i.e., excluding missing observations) in bar charts and tables. For oncological criteria, agreement with existing recommendations was highlighted. Chi-square test was utilized to evaluate associations between background professional factors and experience or attitude regarding CMEC, and the statistical significance was set to *P* < 0.05. IBM SPPS Statistics v28 was used for data analyses.

### Ethical approval

Ethical permission was obtained from the Swedish Ethical Review Authority (Dnr. 2020-06860, decision date 20/01/2021). All participants received written information about the study and participation was voluntary and anonymous. All experiments were performed in accordance with relevant guidelines and regulations. Informed consent was obtained from all participants by survey completion and submission. The study was performed in accordance with the Declaration of Helsinki.

## Data Availability

The datasets generated and/or analysed during the current study are not publicly available due to lack of ethical approval for open data sharing but are available from the corresponding author on reasonable request.
